# Malignant Myeloma in a Patient After Treatment for Osteoporosis with Teriparatide; a Rare Coincidence

**DOI:** 10.4137/ccrep.s1026

**Published:** 2008-08-29

**Authors:** Terje Forslund, Anna-Mari Koski, Arvo Koistinen, Anu Sikiö

**Affiliations:** 1 Division of Nephrology, Department of Medicine, Central Finland Health Care District Hospital, Jyväskylä, Finland.; 2 Division of Endocrinology, Department of Medicine, Central Finland Health Care District Hospital, Jyväskylä, Finland.; 3 Division of Haematology, Department of Medicine, Central Finland Health Care District Hospital, Jyväskylä, Finland.

**Keywords:** multiple myeloma, osteoporosis, parathyroid hormone, teriparatide, renal failure

## Abstract

A breakthrough in understanding of mechanisms of bone structure regulation has brought about the introduction of the new synthetic recombinant human parathyroid hormone 1–34 (PTH1-34; Teriparatide) in the treatment of osteoporosis. These mechanisms, involving the RANKL, RANK, and osteoprotegerin system, are also known to be involved in malignant myeloma (MM) and tumor and bone metastasis development.

We report a case in which MM was found after treatment of osteoporosis with teriparatide. We were unable to demonstrate any direct association between the MM and teriparatide treatment. However, it seemed intriguing that similar mechanisms are activated in the development of MM as those being working during teriparatide treatment.

In the view of our case, we propose that MM by examination of serum protein fraction should be searched for prior to treatment with teriparatide as it is an exclusion criterion in teriparatide treatment of secondary osteoporosis. A search for other metastatic diseases prior to teriparatide treatment should eventually also be considered. The theoretical basis for our proposal is discussed.

## Introduction

The synthetic peptide teriparatide (Forsteo^®^ Europe, Forteo^®^ U.S.A., Eli Lilly and Co., Indianapolis, U.S.A.) which is identical to the N-terminal 1–34 amino acids of endogenous human parathyroid hormone (PTH; rhPTH1-34) has been introduced to treat established osteoporosis in postmenopausal women [1]. Teriparatide treatment effectively reduced the risk of fractures in these subjects [1]. While the effect of PTH on bone is either anabolic or resorptive, intermittent exposure of PTH results in new bone formation due to increased number and stimulation of osteoblasts through PTH receptors. Bone turnover is regulated through the activator of nuclear factor-κB (NF-κB) ligand (RANKL), its receptor RANK, and the protein osteoprotegerin (OPG) (RANKL/OPG) pathway [2]. The action of teriparatide is brought about by intermittent stimulation of PTH receptors on osteoblasts which augments net bone formation. Clinically this is recognized by an initial rise in biochemical markers of osteoblast activity followed by upsurge in markers of osteoclast activity [3]. In osteoblast cell cultures PTH related peptide 1–34 (PTHrP1-34) stimulate the expression of interleukin-6 (IL-6) [2,4].

Osteosarcoma, osteoblast hyperplasia, osteoblastoma, osteoma and fibrosarcoma have been reported in rats given high doses of rhPTH1-34 [5]. Whether osteosarcoma or other malignancies are provoked in humans by rhPTH1-34 is not known, and in the only case so far reported it could not with certainty be concluded that osteosarcoma developed during teriparatide treatment [6]. We report a patient in whom malignant myeloma (MM) that was diagnosed soon after discontinuation of teriparatide treatment, a rare coincidence.

## Case

A female, 57 years-of-age, with heterozygote abnormality in the prothrombin-gene and a 3 to 5 times increased risk of thrombosis underwent total hysterectomy in 1997. Then she had an occipital cerebral infarction from which she recovered with a small central-field blindness. Life-long treatment with warfarin was started. Subsequently, during late 1990’s, she developed osteoporosis of serious degree. Magnetic resonance imagine (MRI) examination in September 2004 demonstrated osteoporosis with compression fractures of several corpora of the lumbar column (LI, LII, LIII, and LIV) and decreased bone mineralization to a lumbar T-score of −3.1 and femoral neck T-score of −1.5. Chronic dorsal pain led to surgery with vertebroplasty of TH 11–12 in May 2005. Unfortunately, an examination of serum protein fraction to exclude a monoclonal component was not performed. Initially she was treated per-orally with bisphosphonates (alendronate and risedronate) which was discontinued due to gastrointestinal side effects. Teriparatide was given subcutaneously (s.c.) at a daily dose of 20 μg from June 2005 and stopped after 18 months in January 2007 as recommended [7]. Addition of calcium and vitamin-D was also given during that period. She had a single dose of 5 mg zoledronic acid in February 2007.

She had normal kidney function in September 2006 with a plasma creatinine (p-creat) concentration of 50 μmol/l. After February 2007, her condition deteriorated rapidly with malaise, nausea, food intolerance and in May 2007 her p-creat concentration had of a sudden increased to 404 μmol/l. Further examinations demonstrated urinary protein excretion of 8 gr/24 hrs, consisting of immunoglobulin kappa light chains (Ig-κ-Lc). Serum Ig-κ-Lc was found in the gamma-fraction, and in the alpha and beta-globulin regions, with suppression of the normal immunoglobulins IgA, IgM and IgG. The amount of serum free Ig-κ-Lc was 21300 mg/l (ref. value 6.9–25.6 mg/l) and that of Lc lambda (λ) was 7.5 mg/l (ref. value 8.6–26.5 mg/l). Further, the β_2_-microglobulin fraction was increased to 23.4 mg/l. Bone marrow examination disclosed 80% of plasma cells verifying the diagnosis of MM. Destructive bone involvement was also found at X-ray examination of humerus, femur and skull. Low serum erythropoietin (EPO) concentration and secondary anemia were present for which human recombinant EPO treatment (NeoRecormon^®^, F. Hoffmann-La Roche Ltd., Basel, Switzerland) was started. Treatment of the MM disease was initiated with vincristine (Oncovin^®^, Pfizer Inc., U.S.A.) and doxorubicin (Adriamycin^®^, Pfizer Inc., U.S.A.) plus intermittent high-dose dexamethasone (VAD).

## Discussion

This is, to our best knowledge, the first report on treatment of osteoporosis with teriparatide with the subsequent discovery of MM. Although primary hyperparathyroidism was demonstrated to co-exist with MM [8,9], no direct association between high levels of circulating PTH and MM have been shown so far. However, benign parathyroid adenoma with persistent hypercalcemia accompanying multiple myeloma was previously reviewed [10]. It has been shown that the RANK, RANKL, OPG system and IL-6 participate in the development of hypercalcemia observed in meta-static prostate and breast carcinoma and in MM bone disease [2,3,9,11]. Others have shown that administration of the RANKL antagonist RANK-Fc limits MM-induced osteoclastogenesis, development of bone disease, and MM tumor progression [12]. Besides stromal/osteoblastic cells, high levels of RANKL expression are restricted to lymphocyte-containing tissues and mammary epithelium [13]. In line with in vivo evidence that RANKL becomes biologically relevant only upon stimulation above of its basal expression, osteoclastogenesis in co-cultures of hematopoietic precursors and stromal/osteoblastic cells occurs only when the latter cell type is stimulated with hormones or cytokines [14,15]. Intermittently given (rhPTH1-34) teriparatide, resulted in stimulation of RANKL and IL-6 protein [2,3,4,16].

Whether or not RANKL is expressed by malignant myeloma cells is still a matter of debate. If not produced directly by these cells, most likely it is produced by marrow stromal cells in response to myeloma cells [16,17]. Experimentally it has been demonstrated that myeloma cell lines could activate stromal cells to express RANKL and inhibit the expression of OPG [18]. In addition, down-regulation of OPG expression in co-cultured pre-osteoblasts and stromal cells was reported [19]. Tumor cells may interact with osteoblasts, thus increasing osteoblast expression of RANKL and decreasing expression of OPG, and may also directly activate osteoclasts by expressing RANKL [2,19]. Activated RANK was shown to protect some hematopoietic neoplastic cells from apoptosis and expression of RANK plays an important role in plasma cell survival in MM as tumor cells directly may activate osteoclasts by expressing RANKL [2]. A recent publication also point out a possible link between hyperparathyroidism and cancer, especially malignant myeloma [20].

The theoretical possibility that osteoporosis treatment with teriparatide could trigger the development of bone disease in pre-existent MM is based in the fact that both teriparatide and indirectly MM may induce the secretion of RANKL [2,3] to amounts higher than that of OPG and thereby disrupting the balance between RANKL and OPG resulting in an increased osteoclast activity. Therefore, teriparatide might be considered an agent that, also when intermittently given, may cause over-stimulation of RANKL which in turn causes activation of osteoclasts and if present enhance MM bone disease. Whether teriparatide, as an extra trigger, may cause such sustained overshoot stimulation of osteoclasts by RANKL in MM patients resulting in osteal destruction in human remains an open question, however.

One might argue that MM existed long before treatment with teriparatide, and that the lumbar fracture actually was caused by MM-associated osteoporosis as described by others [21]. In that report [21] an examination of serum protein fraction had not been performed. Although examination of serum protein fractions to exclude a monoclonal component was not performed prior to teriparatide treatment, it seemed unlikely that MM pre-existed before teriparatide treatment as all other blood tests including hemoglobin, serum calcium, and serum creatinine concentration were normal. Moreover, she had no urinary protein excretion at that point of time. In spite of these results, in our opinion the pathological vertebral fractures seemed unlikely to be due to MM, the possibility of such could not be entirely excluded, however [22]. The RANKL, RANK and OPG system is also involved in the development of metastatic prostatic and breast cancer [2,11]. According to Hodsman et al. [7], there is no reason to believe that treatment with teriparatide would be a significant risk of inducing either bone or non-osseous cancer, but however recommended not to use teriparatide in patients with a history of cancer within the past 5 years. Along with the increasing knowledge on bone mineral turnover and the relation between teriparatide treatment and the RANKL/OPG system [23], it seems clear that measurements of serum RANKL or OPG alone will not be a satisfying tool to identify osteoporosis or to monitor osteoporosis treatment with teriparatide or bisphosphonates [23].

As stated [24] teriparatide is contraindicated in patients with cancer and known bone metastases. In line with previous these recommendations [7,24], as also mentioned for treatment of osteoporosis induced fractures [22,24], we would like to add that patients should be examined for multiple myeloma, and probably also for prostatic and mammary cancer prior to the initiation of teriparatide treatment. The presence of benign monoclonal gammopathy may occasionally also develop to malignant gammopathy. This case, the first to describe the co-existence of MM and osteoporosis treated with teriparatide, does not justify the conclusion that teriparatide is implicated in the development of MM, a rare co-incidence, however.
